# Screening and Identification of Soil Selenium-Enriched Strains and Application in *Auricularia auricula*

**DOI:** 10.3390/microorganisms12061136

**Published:** 2024-06-02

**Authors:** Yadong Chen, Zhenghan Liu, Weimin Zeng, Yang Liu, Dandan Zhao, Yanlong Zhang, Xiangqian Jia

**Affiliations:** 1 Engineering Research Center of Agricultural Microbiology Technology, Ministry of Education & Heilongjiang Provincial Key Laboratory of Ecological Restoration and Resource Utilization for Cold Region & Key Laboratory of Microbiology, College of Heilongjiang Province, School of Life Sciences, Heilongjiang University, Harbin 150080, China; yadong991125@163.com (Y.C.); liu326331277@163.com (Z.L.); 2002081@hlju.edu.cn (W.Z.); 2021103@hlju.edu.cn (Y.L.); zhaodandan@hlju.edu.cn (D.Z.); 2 Post-Doctoral Scientific Research Workstation of Heilongjiang Boli Economic Development Zone Management Committee, Qitaihe 154500, China

**Keywords:** Se, bacterial strain, *Enterobacter*, organic Se, *Auricularia auricula*

## Abstract

Selenium (Se) is an essential trace element for human physiological metabolism. The application of organic Se as a source to cultivate Se-rich plants for micronutrient supplementation has been receiving increasing attention. In our study, a bacterial strain named H1 was isolated from the soil in Heilongjiang Province, China, and under optimal culture conditions, the unit Se content could reach 3000 μg·g^−1^ and its 16S ribosomal DNA sequence seemed to be a new molecular record of an *Enterobacter* species. After the domestication of Se tolerance and Se-rich experiments, H1 can be used as a Se source for cultivation of Se-rich *Auricularia auricula*. The results showed that soluble protein, soluble sugar, free amino acid and vitamin C contents in *Auricularia auricula* were notably increased by 28.7%, 21.8%, 32.5% and 39.2% under the treatment of Se concentration of 0.24 mg·kg^−1^, respectively. These findings enhance our understanding that H1 is more conducive to Se uptake and nutrient accumulation.

## 1. Introduction

Selenium (Se) is an essential element for human health. It has been found in recent years to maintain the normal function of the cardiovascular system, improve immunity, detoxify the body and protect the heart [1,2]. Se deficiency may damage the nervous system, reduce the immune system and cause Kashin–Beck disease [3,4]. Thus, appropriate Se intake is significant for human health, such as for enhancing the activity of natural killer cells and inhibiting the growth of cancer cells [5].

Se can be supplemented through many ways, generally using inorganic Se and organic Se. Compared to inorganic Se, organic Se has lower toxicity, high bioavailability and low environmental pollution [6,7]. To convert inorganic Se into organic Se, biotransformations including plant transformation, animal transformation and microbial transformation are mainly carried out at present [8]. Microorganisms, including bacteria, fungi and other biological groups, are most commonly used as Se carriers due to their fast growth, hypermetabolism, strong vitality and high yield. Microorganisms play an important role in the chemical cycle of Se, which mainly realizes the conversion of inorganic Se to organic Se through the homocysteine synthase pathway and cysteine synthase pathway [9,10,11]. In microorganisms, bacteria are the most abundant group, and their tolerance to Se are greater than fungi and algae. Ullah et al. identified a high-Se strain, Bacillus subtilis BSN313, from Chinese traditional liquor starter, capable of accumulating Se up to 2123 μg·g^−1^ [12]. Martínez et al. conducted Se-rich experiments with 96 strains and found that *Lactobacillus*, *Enterococcus* and other bacteria could achieve a conversion rate of 24–50% for inorganic selenium [9]. Pieniz et al. investigated the Se-rich capacity of *Enterococcus*. They subjected the biomass obtained from the strains to dialysis to remove inorganic Se and determined the content and distribution of organic Se in the strains. The results revealed that the proportion of organic Se was highest in the protein fraction, followed by polysaccharides and nucleic acids [13]. Therefore, using bacteria to enrich inorganic Se can improve the value of microorganisms and provide a reference for the utilization of Se resources to produce Se-rich agricultural products [14,15]. Therefore, the screening of strains with high Se tolerance and strong transformation ability has broad development prospects in resource utilization, Se-rich food and Se-rich agricultural products.

In recent years, with people’s demand for health, the Se-rich agricultural products industry has been rapidly developed [16,17]. For example, Yuan et al. used organic Se fertilizer to spray rice, resulting in safe and green Se-rich rice [18]. Wu et al. found that spraying Se fertilizer significantly increased the free amino acid and total protein content of Cardamine violifolia and enhanced the quality of Cardamine violifolia [19]. Se has an important regulatory effect on the growth of mushroom microorganisms. Edible fungi such as *Auricularia auricula* and shiitake mushrooms can enrich Se. However, currently, inorganic Se sources such as sodium selenite are generally used domestically and internationally, and there is limited research on organic Se sources. In this context, due to low toxicity, easy absorption, and no damage to soil structure, organic Se fertilizers are widely used as Se-rich supplements in the cultivation of agricultural products. Hence, the development of new organic Se bacteria fertilizer is of great significance in improving the quality of agricultural by-products and fertilizing the soil. *Auricularia auricula* is a precious colloidal fungusand, and it is also the fourth largest cultivated mushroom species in the world. *Auricularia auricula* is rich in protein, polysaccharides, vitamins and trace elements, and has many effects such as lowering blood lipids, anti-tumor and anti-inflammation. Because of its unique nutritional and medicinal value, *Auricularia auricula* has attracted much attention in Asia and even around the world [20,21]. The current research on Se-rich *Auricularia auricula* mainly focuses on two aspects of using inorganic Se added to the cultivation substrate or spraying an inorganic Se solution on the bacterial packet, but because inorganic Se has a large toxicity, the Se-rich effect of *Auricularia auricula* is not ideal. In addition, the application of Se-rich bacteria to the cultivation of Se-rich *Auricularia auricula* is rarely reported. This phenomenon inspired us to explore novel Se-rich bacteria.

**Figure 1:**
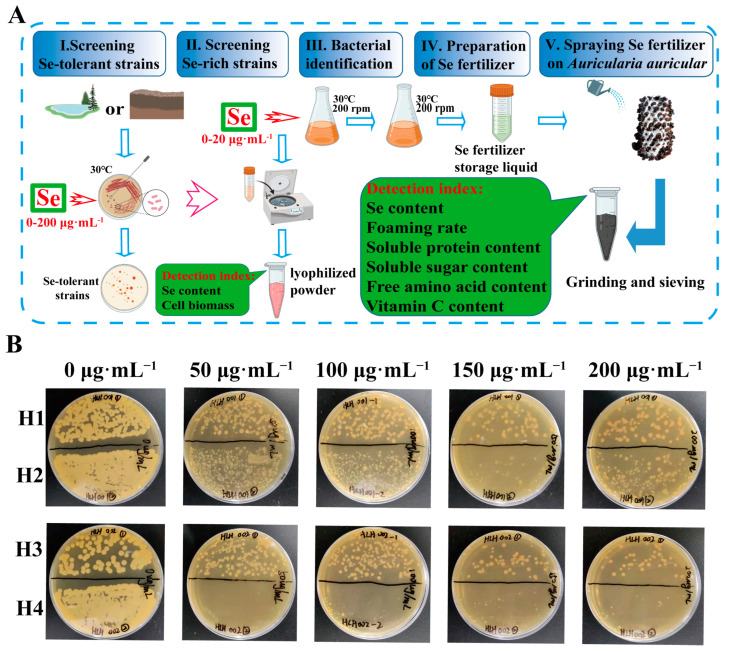
(**A**) Schematic diagram of the experimental procedure. (**B**) Growth of strains (H1, H2, H3 and H4) from dry fields on medium containing high concentration of Se.

Hailun (Heilongjiang Province, China) has Se-rich dry fields and paddy fields. In this work, we chose Changhua Village as a sampling site to explore potential bacterial strain and isolated a novel bacterial strain H1. Briefly, the experiment consists of five steps (Figure 1A): screening Se-tolerant strains, screening Se-rich strains, bacterial identification, preparation of Se fertilizer, and spraying Se fertilizer on *Auricularia auricula*. The strain H1 was used as Se fertilizer raw material after ultrasonic crushing. The soluble protein, soluble sugar, free amino acid and vitamin C contents in *Auricularia auricula* were notably increased, which suggest that this study will provide new insights into Se-rich *Auricularia auricula* cultivation.

## 2. Materials and Methods

### 2.1. Isolation and Cultivation of Se-Tolerant Strains

Hailun City, Heilongjiang Province (126°51′ E 47°32′ N), China, was selected as the sampling site to isolate bacterial strains. Three soil samples of 500 g each were collected from the upper (1−10 cm), middle (10−20 cm) and lower (20−30 cm) layers. The preparation method of bacterial suspension refers to the research of Thomas et al. [22]. Briefly, 1 mL of the bacterial suspension were inoculated into 20 mL of sterilized liquid medium (glucose 20 g·L^−1^, yeast extract powder 10 g·L^−1^, peptone 20 g·L^−1^, pH 6.0) and incubated for 48 h with rotation speed 200 rpm, temperature 30 °C. The limit of tolerance to Se refers to the research of Ullah et al. [12]. Briefly, the inoculated liquid was plated onto solid medium with sodium Se content of 0, 50, 100, 150 and 200 μg·mL^−1^, which was kept at 30 °C until single colonies appeared. Se-tolerant colonies were picked and purified by restriping on plates. The purified strains were confirmed by microscopic examination and repeated scribing on the plate.

### 2.2. Se-Tolerant Strain Growth and Se-Bioaccumulation

In order to better convert inorganic Se into organic Se, the high Se-rich strains were selected from the Se-tolerant strains. A volume of 1 mL of different Se-tolerant strains suspension were inoculated into 20 mL of sterilized liquid medium with sodium selenite content of 0, 5, 10, 15 and 20 μg·mL^−1^ and incubated for 36 h with rotation speed 200 rpm, temperature 30 °C. Then the Se-rich strains were determined by measuring the biomass, unit Se content and total Se content of fermentation [23].

### 2.3. Determination of Biomass and Se Content

The biomass of the Se-rich strains was determined with reference to Mörschbächer et al. [24]. The unit Se content of Se-rich strains was determined as described by Chand et al. and Zou et al. [25,26]. In brief, 0.005 g of samples were weighed and placed in a digestion tube, subsequently digested with 1 mL of nitric acid–perchloric acid (*v*/*v*, 4:1) mixture solution, cold digestion overnight, and the next day were heated on the heating plate, until the solution became colorless and produced a large amount of white smoke. After cooling to room temperature, deionized water was added to the sample to facilitate the assay and the volume was fixed to 5 mL. The Se content was determined and expressed as μg·g^−1^. The total fermentation Se content (μg·L^−1^) of the strain was calculated using the following equation.

Total Se content in fermentation (μg·L^−1^) = Unit Se content (μg·g^−1^) × Cell biomass (g·L^−1^)

### 2.4. Amplification and Sequencing of 16S rDNA Sequence

The total genomic DNA of Se-rich strains cells was extracted using a DNA extraction kit (Beijing Solarbio Science & Technology Co., Ltd., Beijing, China) and was sent to Suzhou Jinweizhi Biotechnology Co., Ltd. (Suzhou, China) for 16S rDNA amplification and amplification product sequencing.

### 2.5. Phylogenetic Analysis

To obtain phylogenetic information of strain H1, its 16S rDNA sequence was used as query sequence to search for similar sequences in the NCBI GenBank database. Using BLAST to obtain 10 sequences with high similarity to H1, phylogenetic reconstruction and validation were performed using the NJ method based on bootstrap analysis with 1000 replications using Jukes–Cantor distance model [27]. Escherichia coli ATCC11775 (TX80725) was chosen as an outgroup to determine the root of the phylogenetic tree. This sequence was repeatedly compared to additional selected sequences from the same strain family in the GenBank database using the ClustalW methodology. Using MEGA 7.0, a phylogenetic tree was constructed based on neighborhood linkage [28].

### 2.6. Application in the Cultivation of Auricularia auricula

“Jinfeng” *Auricularia auricula* is a promoted planting variety in Heilongjiang Province. It has excellent characteristics such as good ear shape and neat edges of ear pieces. “Jinfeng” *Auricularia auricula* in Boli County, Qitaihe City, Heilongjiang Province (130°35′ E 45°45′ N) was selected as the treatment group with three replicates. Three treatment groups were set up in the experiment, with a control group (CK) sprayed with water. Each *Auricularia auricula* bag was sprayed with 40 mL of the assigned treatment. The commercial Selenium fertilizer group (A) was purchased in the market (Se fertilizer name is “Zhen Xi”, from Guilin Jiqi Biochemical Co., Ltd., Guilin, China), diluted 200 times according to the instructions. At this time, the Se mass concentration was 0.24 mg·kg^−1^, and each *Auricularia auricula* bag was sprayed with 40 mL of the commercial Selenium fertilizer. The experimental groups (B1: Se mass concentration 0.18 mg·kg^−1^, B2: 0.24 mg·kg^−1^, B3: 0.36 mg·kg^−1^, B4: 0.72 mg·kg^−1^, each *Auricularia auricula* bag: 40 mL) were sprayed with our Se fertilizer, which was the cracking substance of H1. Nine days after the transplant, *Auricularia auricula* were measured. The *Auricularia auricula* were dried in a natural environment, crushed and then passed through a 100-mesh sieve for the determination of Se, soluble sugar, soluble protein, vitamin C and free amino acid contents.

### 2.7. Determination of Total Se Content in Auricularia auricula

The total Se content in *Auricularia auricula* was determined by the method described by Chand et al. and Zou et al. [25,26]. In brief, 0.005 g of samples were weighed and placed in a digestion tube, subsequently digested with 1 mL of nitric acid–perchloric acid mixture solution, cold digestion overnight, and the next day were heated on the heating plate, until the solution became colorless and produced a large amount of white smoke. After natural cooling, the volume was fixed to 5 mL with deionized water, and the Se content was determined.

### 2.8. Determination of Foaming Rate of Auricularia auricula

We selected an *Auricularia auricula* fruiting body with a certain quality, dried it in a drying oven at 50 °C for 24 h, weighed it, and recorded it as A. We also soaked a fruiting body in 2 L of ultrapure water, allowing it to absorb water to a constant weight, then removed surface moisture, weighed it, and recorded it as B.

Auricularia blister rate = B:A

### 2.9. Determination of Soluble Sugar in Auricularia auricula

The content of soluble sugar was determined by anthrone colorimetry [29]. We weighed 0.05 g of samples and placed it in a dry 10 mL graduated test tube and heated the water bath for 30 min. Then, the solution was centrifuged at 4 °C and 8000 rmp for 10 min and the supernatant was collected. After that, 0.5 mL of the supernatant was mixed with 1.5 mL of distilled water, 0.5 mL of anthrone ethyl acetate reagent and 5 mL of concentrated sulfuric acid in a new dry test tube, shaken quickly and well, then cooled to room temperature. Finally, soluble sugars were measured at a wavelength of 620 nm using a UV spectrophotometer (752 N, Shanghai Yi electric Analytical Instrument Co., Ltd., Shanghai, China). The experiments contained three biological replicates.

### 2.10. Determination of Soluble Protein in Auricularia auricula

The content of soluble protein was determined as previously described by Turfan et al. [30]. A total of 0.1 g of samples were homogenized with 5 mL of distilled water and ultrasonically extracted at 30 °C for 30 min. The solution was then centrifuged at 4 °C and 8000 rmp for 10 min to collect the supernatant. The protein content was determined using the BCA reagent kit (Beijing Solarbio Science & Technology Co., Ltd., Beijing, China). The experiments contained three biological replicates.

### 2.11. Determination of Free Amino Acid and Vitamin C in Auricularia auricula

The content of free amino acids was determined by the ninhydrin colorimetric method [31]. The free amino acid was evaluated by an amino acid content detection kit (Beijing Solarbio Science & Technology Co., Ltd., Beijing, China). Vitamin C was determined by the 2,6-dichlorophenol-indophenol titration method and detected by microplate reader (Bio-Rad, USA) at a wavelength of 525 nm. Each experiment contained three biological replicates.

### 2.12. Statistical Analysis

The results were statistically analyzed by SPSS 17.0 via one-way analysis of variance following Duncan’s multiple significant difference test (*p* ≤ 0.05). Data were recorded as mean ± SD. Graphs were plotted using Graphpad Prism 8.0.2.

## 3. Results and Discussion

### 3.1. Isolation of Se-Tolerant Strain

The experimental samples were collected from dry fields and paddy fields located in Hailun City, Heilongjiang Province, China. The fields in Hailun could be a potent source of Se-rich strains, as there are 12.67 million m^2^ of concentrated Se-rich land there. The average content of Se in soil is 0.40 mg·kg^−1^, and the highest can reach 0.73 mg·kg^−1^, which is the dominant area for Se-rich product development. Therefore, it is expected that we may isolate new Se-rich strains from these areas. A total of 10 bacterial strains were isolated from the samples. The growth of bacterial strains during the different phases of culture in the presence of 0, 50, 100, 150 and 200 μg·mL^−1^ of Se are shown in Figure 1B and Figure S1. The resistance of all strains to Se are different. Six strains (named S1–S6) from the paddy field could not survive high concentrations of Se (Figure S1). Strain H4 isolated from the dry field did not grow on the medium containing 50 μg·mL^−1^ sodium selenite. In contrast, strains H1, H2 and H3 isolated from the dry field could still grow on medium containing 200 μg·mL^−1^ sodium selenite and had good Se tolerance (Figure 1B). Therefore, we selected strains H1, H2 and H3 for the Se bioaccumulation test.

### 3.2. Screening of Optimal Se-Rich Bacteria

In order to screen the strains with strong Se-rich ability, it is necessary to calculate the fermentation total Se content of the strains. At this point, it is necessary to determine the cell biomass and unit Se content of the strain. Table 1 shows the production of cell biomass and unit Se content in the three strains during the growth test under different Se concentration after cell drying. The presence of Se in the medium significantly changed the cell biomass and unit Se content of three strains. Currently, there are many reports on the study of Se-rich strains. For example, the study of Kousha et al. showed that the enrichment ability of *Pediococcus acidilactici* on Se could reach 1450 μg·g^−1^ [32], while Ullah et al. showed that the enrichment ability of *Bacillus subtilis* BSN313 Se could reach 2123 μg·g^−1^ [12]. Under the optimal Se concentration conditions, our three strains were able to achieve a unit Se content of up to 3000 μg·g^−1^ (Table 1). However, when the Se concentration in the medium reached 20 μg·mL^−1^, the biomass of the strain showed a decreasing trend. This may be due to the increase in toxicity caused by the increase in Se concentration in the medium, which produced reactive oxygen species that interfered with the normal growth of the strain [33]. Zhang et al. found that the inhibitory effect of different concentrations of Se on the growth of the strain may be related to the strain type and antioxidant system [10].

**Figure 2:**
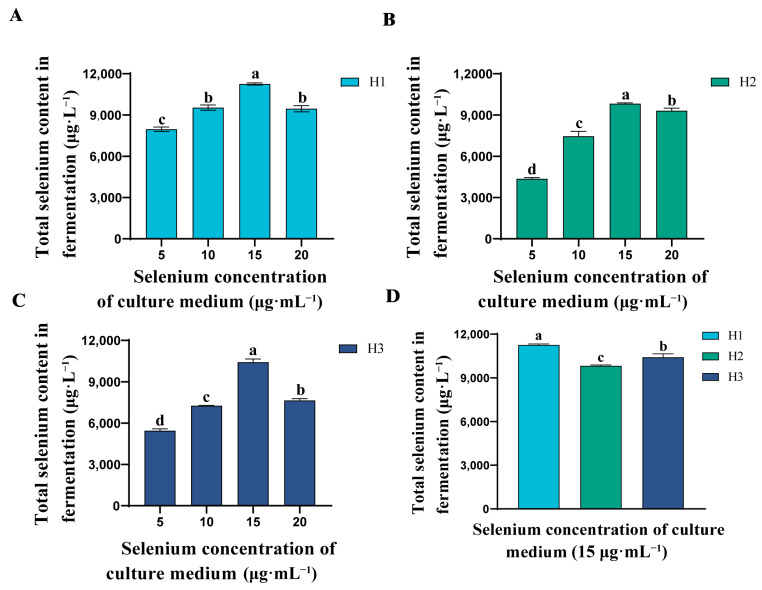
Comparison of total Se content in fermentation of three strains. (**A**) Total Se content in fermentation of strain H1 under different Se concentrations. (**B**) Total Se content in fermentation of strain H2 under different Se concentrations. (**C**) Total Se content in fermentation of strain H3 under different Se concentrations. (**D**) The total Se content in fermentation of strains H1, H2 and H3 at Se concentration of 15 μg·mL^−1^. Values are mean ± standard deviation. The bars represent the mean ± SE, n = 3 replicates. The a, b, c, and d lettering indicates differences between treatments (*p* < 0.05).

In order to investigate which of the three strains has a higher Se-rich capacity, we calculated the Se-rich capacity of the three strains when incubated with different concentrations of inorganic Se. Figure 2A–C show that the total Se content of the three strains increased with the increase of inorganic Se concentration in the medium and reached the highest value at 15 μg·mL^−1^. However, when the inorganic Se concentration was 20 μg·mL^−1^, the total Se content of the strains fermented showed a decreasing trend. This may be due to the fact that high inorganic Se concentrations lead to the destruction of proteins and loss of their original biological activity, thus inhibiting cell growth [34]. From Figure 2C, it is shown that H1 has better Se-rich capacity than H2 and H3 at a Se incubation concentration of 15 μg·mL^−1^. Therefore, we selected H1 as the best Se-rich strain for strain identification and used it to prepare the organic Se fertilizer.

### 3.3. Identification of Strains

**Figure 3:**
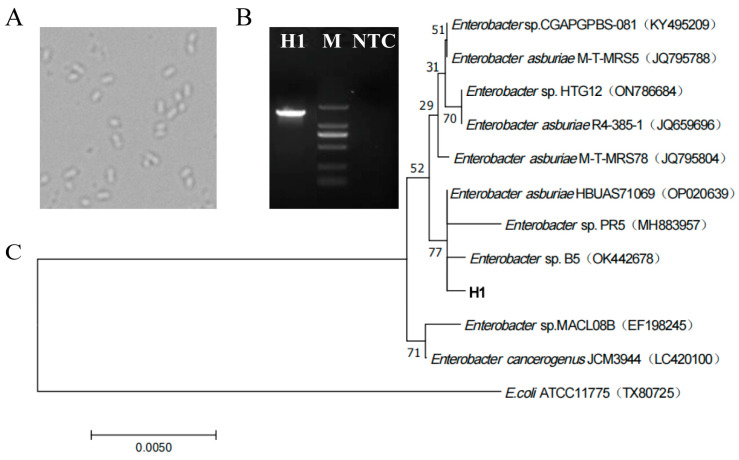
Identification of strains. (**A**) Microscopic morphology of strain H1, 40×. (**B**) PCR product images of strain H1 were detected by agarose gel electrophoresis, M: Marker (DL2000: 2000, 1000, 750, 500, 250, 100 bp), NTC: Negative control. (**C**) According to the phylogenetic tree constructed by BLAST homology search results, E.coli ATCC11775 (TX80725) was selected as the exogenous bacteria to determine the root of the phylogenetic tree. The statistical reliability of the tree topology was confirmed by bootstrapping 1000 resamples of the sequence. The bootstrap values are displayed on the nodes of the tree.

Under the optical microscope (Figure 3A), H1 cells grew freely, and the cell morphology was short-rod-shaped with uniform morphology. The length of 16S rDNA of strain H1 was 1416 bp, which was successfully amplified by PCR (Figure 3B) and sequenced. The sequence has been deposited in GenBank with accession number OP389203. According to the BLAST homology search GenBank database display and phylogenetic tree analysis (Figure 3C), it was found that all other strains belonged to *Enterobacter* (except for the exogenous bacteria). The 16S rDNA sequence of strain H1 was consistent with that of eight strains (four *Enterobacter asburiae* strains: JQ795788, JQ659696, JQ795804 and OP020639; four unknown species of *Enterobacter*: KY495209, ON786684, MH883957 and OK442678) and had a relatively close relationship. Especially regarding the strains *Enterobacter asburia* HBUAS71069 (OP020639), *Enterobacter* sp. PR5 (MH883957) and *Enterobacter* sp. B5 (OK442678), they are in the same branch as strain H1. Among the three strains, *Enterobacter* sp. PR5 (MH883957) and *Enterobacter* sp. B5 (OK442678) are unknown species. The difference between them may be the cause of lower branch confidence (77%). When querying the NCBI database using the 16S rDNA sequence of the H1, through BLAST comparison, it was found that there was no complete sequence match with strain H1 in the NCBI database, and H1 had great similarity with *Enterobacter asburiae* HBUAS71069 (OP020639), *Enterobacter* sp. PR5 (MH883957) and *Enterobacter* sp. B5 (OK442678). Considering that the phylogenetic tree could not identify strain H1 at the species level, the strain was named *Enterobacter* sp. H1, and its 16S rDNA sequence information appeared to be a new molecular record for *Enterobacter* species.

### 3.4. Effect of Spraying Se Fertilizer on the Se Content of Auricularia auricula

**Figure 4:**
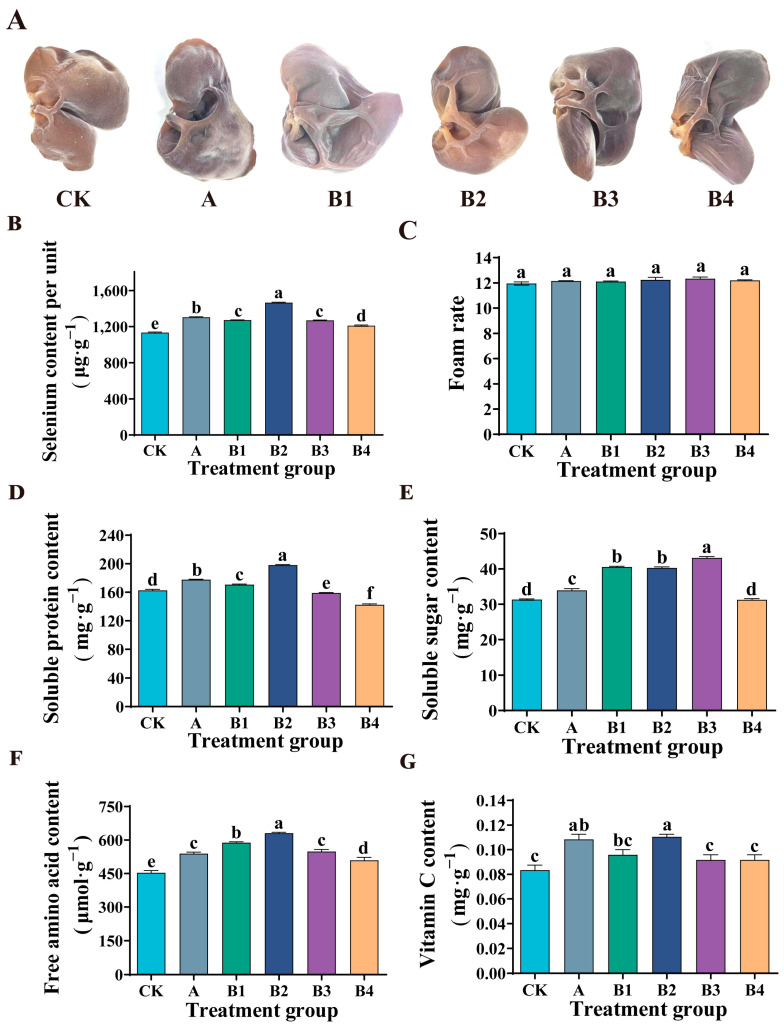
Effect of Se fertilizer on *Auricularia auricula* quality. (**A**) Phenotypes. (**B**) Content of Se per unit. (**C**) Foaming rate. (**D**) Soluble protein content. (**E**) Soluble sugar content. (**F**) Free amino acid content. (**G**) Vitamin C content. In the figure, CK is the control group, A is the commercial Se fertilizer group, and B1, B2, B3 and B4 are the experimental groups with Se mass concentrations of 0.18, 0.24, 0.36 and 0.72 mg·kg^−1^, respectively. Different lowercase letters indicate significant differences (*p* < 0.05) between the two groups.

As shown in Figure 4B, in the experimental group (B1, B2, B3, B4), with the increase of Se concentration, the Se content of *Auricularia auricula* increased significantly. When the Se concentration reached 0.24 mg·kg^−1^, the Se content of *Auricularia auricula* reached the highest (1466.9 ± 6.16 μg·g^−1^), which was 29.3% higher than that of the control group (CK). When the mass concentration of Se exceeded 0.24 mg·kg^−1^, the Se content of *Auricularia auricula* showed a downward trend, but it was still significantly higher than that of the control group (CK). Moreover, compared with the commercial Se fertilizer group (A), at the same Se concentration (0.24 mg·kg^−1^), the Se content in *Auricularia auricula* is higher. The results showed that spraying organic Se fertilizer was helpful to the accumulation of Se in *Auricularia auricula* and could improve the quality of *Auricularia auricula*.

### 3.5. Effects of Spraying Se Fertilizer on Foaming Rate, Soluble Protein and Soluble Sugar Content of Auricularia auricula

In this study, Se fertilizer sprayed by the commercial Se fertilizer group (A) and experimental groups (B1, B2, B3, B4) had no effect on the foaming rate of *Auricularia auricula* (Figure 4C). The experimental group (B1, B2, B3, B4) showed that the soluble protein content in *Auricularia auricula* increased first and then decreased with the increase of Se concentration (Figure 4D). Under low Se treatment (0.18 mg·kg^−1^, 0.24 mg·kg^−1^), the soluble protein content of *Auricularia auricula* increased significantly, which was 4.9% and 21.8% higher than that of the control group, respectively. Ulhassan et al. also showed similar results in the study of Brassica napus [29]. The increase of soluble protein content may be due to the absorption of organic Se in Se fertilizer by *Auricularia auricula* in the form of amino acids, thus regulating the synthesis of protein. Similarly, the results of Handa et al. are also the same [31]. The application of Se increased the content of protein and nitrogen in mustard seedlings, and this explains the direct effect of Se metabolism on nitrogen metabolism. Under high Se treatment (0.36 mg·kg^−1^, 0.72 mg·kg^−1^), the soluble protein content of *Auricularia auricula* decreased significantly. This may be due to the excess of Se, which leads to the substitution of sulfur in some amino acids by Se, and the destruction of protein structure, thus reducing the accumulation of protein [35]. In addition, compared with the commercial Se fertilizer group (A), the soluble protein content in *Auricularia auricula* was also higher under the same Se mass concentration (0.24 mg·kg^−1^).

Compared with the control group, the application of commercial Se fertilizer significantly increased the soluble sugar content of *Auricularia auricula* (Figure 4E), but the advantage was not significant compared to the experimental group. In the experimental group, compared with the control group, the content of soluble sugar in *Auricularia auricula* increased by 29.5%, 28.7% and 37.7%, respectively, when the Se concentration was 0.18, 0.24 and 0.36 mg·kg^−1^ (Figure 4E). That is similar to the research results of Liao et al. on Se-rich cabbage and of Hajiboland et al. on Se-rich Brassica napus [36,37]. This indicated that spraying Se fertilizer could induce the accumulation of soluble sugar and improve the nutritional quality of *Auricularia auricula*.

### 3.6. Effects of Se Fertilizer on the Contents of Free Amino Acids and Vitamin C in Auricularia auricula

Amino acids are precursors of numerous secondary metabolites. After spraying Se fertilizer, the content of free amino acids of *Auricularia auricula* in the commercial Se fertilizer group and experimental group was significantly increased (Figure 4F). Similarly, compared with the experimental group, under the same Se mass concentration (0.24 mg·kg^−1^), the content of free amino acids in *Auricularia auricula* in the experimental group was more advantageous. The experimental group showed that when Se concentration was 0.18, 0.24, 0.36 and 0.72 mg·kg^−1^, the free amino acid content of *Auricularia auricula* was increased by 29.8%, 39.2%, 21% and 12.3%, respectively. The results indicate that Se fertilizer was beneficial to the accumulation of free amino acids in *Auricularia auricula*. Moreover, this is consistent with the application of Se in mustard seedlings reported by Handa et al. [31]. These results may be due to the fact that Se metabolized in *Auricularia auricula* is mainly in the form of amino acids.

Vitamin C is a compound with high biological activity. In our study, the application of commercial Se fertilizer significantly increased the vitamin C content of *Auricularia auricula*. Moreover, spraying our Se fertilizer was more effective compared to commercial selenium fertilizers at the same Se mass concentration (0.24 mg·kg^−1^) (Figure 4G). When the Se concentration in the experimental group was 0.24 mg·kg^−1^, the content of vitamin C in *Auricularia auricula* was 32.0% higher than that in the control group. Gui et al. found a similar trend in their study on broccoli, which may be due to the fact that Se can increase Se-protein in broccoli, thereby reducing the oxidation of vitamin C by intracellular hydrogen peroxide [38]. In addition, the amino acid itself may promote vitamin C accumulation.

## 4. Conclusions

In order to explore strains with high Se-rich ability, a Se-rich strain named H1 was isolated from Se-rich soil in Hailun City, China. After morphological characterization and phylogenetic analysis, strain H1 was named *Enterobacter* sp. H1. The Se fertilizer was prepared by this strain and sprayed on *Auricularia auricula*. The results showed that the Se fertilizer could increase the Se content of the fruiting body of *Auricularia auricula* and improve the nutritional quality of *Auricularia auricula*. The above results are of great significance for the development of new Se-rich strains and the development of organic Se fertilizers.

## Figures and Tables

**Table 1:** Comparison of cell biomass and unit Se content of strains under different Se concentration conditions.

	Strain	Se Concentration (μg·mL^−1^)				
		0	5	10	15	20
Cell biomass (g·L^−1^)	H1	2.83 ± 0.02 ^d^	2.84 ± 0.01 ^d^	3 ± 0.02 ^b^	3.32 ± 0.00 ^a^	2.95 ± 0.03 ^c^
	H2	3.05 ± 0.00 ^b^	2.1 ± 0.02 ^e^	2.79 ± 0.04 ^d^	3.28 ± 0.02 ^a^	2.88 ± 0.01 ^c^
	H3	3.11 ± 0.01 ^b^	2.21 ± 0.02 ^d^	2.22 ± 0.02 ^d^	3.25 ± 0.02 ^a^	2.41 ± 0.03 ^c^
unit Se content (μg·g^−1^)	H1	2383.9 ± 30.1 ^d^	2802.9 ± 56.0 ^c^	3179.9 ± 64.2 ^b^	3389.9 ± 22.7 ^a^	3203.9 ± 75.4 ^b^
	H2	2142.9 ± 8.0 ^d^	2076.9 ± 39.0 ^d^	2663.9 ± 53.1 ^c^	2993.9 ± 20.0 ^b^	3226.9 ± 63.0 ^a^
	H3	2664.9 ± 18.7 ^b^	2466.9 ± 61.3 ^c^	3268.9 ± 10.6 ^a^	3204.9 ± 71.7 ^a^	3187.9 ± 54.4 ^a^

The bars represent the mean ± SE, n = 3 replicates.The a, b, c, d, and e lettering indicates differences between treatments (*p* < 0.05).

## Data Availability

Data are contained within the article and Supplementary Materials.

## Supplementary Materials

The following supporting information can be downloaded at: https://www.mdpi.com/article/10.3390/microorganisms12061136/s1, Supplementary Data: The sequence of 16S rDNA of strain H1; Figure S1: Screening of Se-tolerant strains: Six strains numbered S1, S2, S3, S4, S5 and S6 were isolated from paddy fields.
